# Proteomic Dynamics in the Interaction of Susceptible and Resistant Tomato Cultivars and Potato Cyst Nematodes

**DOI:** 10.3390/ijms26062823

**Published:** 2025-03-20

**Authors:** Marek D. Koter, Marek Żurczak, Mateusz Matuszkiewicz, Magdalena Święcicka, Maciej Kotliński, Anna Barczak-Brzyżek, Marcin Filipecki

**Affiliations:** 1 Department of Plant Genetics, Breeding and Biotechnology, Institute of Biology, Warsaw University of Life Sciences—SGGW, Nowoursynowska 159, 02-776 Warsaw, Poland; marek_koter@sggw.edu.pl (M.D.K.); mateusz_matuszkiewicz@sggw.edu.pl (M.M.); magdalena_swiecicka@sggw.edu.pl (M.Ś.); anna_barczak-brzyzek@sggw.edu.pl (A.B.-B.); 2 Department of Systems Biology, Institute of Experimental Plant Biology and Biotechnology, University of Warsaw, Pawińskiego 5A, 02-106 Warsaw, Poland; m.kotlinski@uw.edu.pl

**Keywords:** proteomics, nematode resistance, *Solanum lycopersicum*, *Globodera rostochiensis*, *Arabidopsis thaliana*, *Heterodera schachtii*

## Abstract

This study investigates the proteomic dynamics in tomato cultivars with differing resistance to potato cyst nematodes (PCNs). Cyst-forming nematodes, significant agricultural pests, induce complex molecular responses in host plants, forming syncytia in roots for their nutrition. This research employs mass spectrometry to analyze the proteomes of infected and uninfected roots from susceptible (Moneymaker) and resistant (LA1792 and L10) tomato lines. Over 2800 high-confidence protein hits were identified, revealing significant differences in abundance between susceptible and resistant lines. Notably, resistant lines exhibited a higher number of newly expressed proteins compared to susceptible lines; however, the proportion of induced and suppressed proteins was strongly genotype-dependent. Gene ontology (GO) analysis highlighted that nematode infection in susceptible line significantly regulates many defense-related proteins, particularly those involved in oxidative stress, with a similar number being upregulated and downregulated. Some GO terms enriched among nematode-regulated proteins also indicate the involvement of programmed cell death (PCD)-related processes. The susceptible line exhibited a prevalence of downregulated proteins, among which defense associated GO terms were significantly overrepresented. Four proteins (APY2, NIA2, GABA-T, and AATP1) potentially crucial for nematode parasitism were identified and their *Arabidopsis* orthologs were studied. Mutant *Arabidopsis* lines showed altered nematode resistance, supporting the involvement of these proteins in plant defense. This study highlights the complexity of host-nematode interactions and emphasizes the importance of proteomic analyses in identifying key factors and understanding plant defense mechanisms.

## 1. Introduction

Cyst-forming plant parasitic nematodes are severe pests in agriculture and, at the same time, an exceptional model for studying the molecular interplay between representatives of two kingdoms. These tiny parasites are responsible for average worldwide crop losses estimated at between US $118 billion (based on 40 crops in the 2001 season) [1,2] and US $358 billion (based on 37 crops from the 2010–2013 seasons) [3] per year. Besides relatively unspecific symptoms on the aboveground part—weaker growth and wilting resembling nutrient and water deficit—nematodes evoke surprisingly complex molecular reactions of susceptible hosts. A closer look at the roots reveals small cysts, the dead females filled with eggs attached to locally swelled roots. These swellings are caused by specific multinucleate root structures called syncytia designed to feed developing nematode larvae and adults [4]. The root cells must be deeply reprogrammed to form these hyperplastic and hypermetabolic syncytia and maintain their feeding function.

Syncytia are initiated by infective juveniles (J2), which hatch from eggs (the first molt is within the egg) and attack new roots, migrating intracellularly to localize the initial syncytial cell (ISC), usually within the vascular cylinder. The nematode attack and migration are facilitated by mechanical action of stylet and protein secretions from subventral esophageal glands. The dorsal gland secretions are, in turn, responsible for transforming ISC into a functional syncytium [5]. The syncytium grows by integrating the ISC with vascular cylinder cells, forming large cell wall dissolutions [6,7]. The syncytial cytoplasm becomes dense with an increased number of organelles and vesicles [6,7]. Moreover, the syncytium increases in volume due to elevated turgor pressure, which results from higher osmolyte content and symplasmic isolation from surrounding tissues [8].

The nematode secretions contain a diverse set of effectors that target various cellular processes involved in syncytium development, including cell wall modification, signal transduction, phytohormonal homeostasis [9,10,11,12], as well as pre-mRNA splicing, potentially modifying all cellular functions [13]. However, the core group of effectors is directly engaged in host defense suppression. For example, the *Globodera rostochiensis* (Potato Cyst Nematode, PCN) effector VAP1 (Venom allergen-like protein) interacts with the host papain-like cysteine protease Rcr3pim to reduce plant immune response, and the SPRYSEC protein interacting with SW5 protein family containing CC-NB-LRR domain [14,15]. More recently, the GrUBCEP12 and GrEXPB2 effectors were discovered to suppress immune signaling induced in the cytoplasm [16].

Defense responses, as well as developmental and metabolic reprogramming of root cells during syncytium formation and functioning, are extensively visualized by global changes in gene expression. Various techniques have been used to study transcriptome changes during syncytium development in model *Arabidopsis* and crop plants. Swiecicka and colleagues [17] used cDNA-AFLP to study tomato response to *G. rostochiensis* parasitism and described 265 differentially expressed genes. In *H. schachtii*-infected *Arabidopsis* roots, transcriptome dynamics was visualized using microarray hybridization of cDNAs deriving from microaspirated RNA from syncytia, revealing 3893 up-regulated and 3338 down-regulated genes at 5 and 15 days post-infection [18]. NGS-based techniques have also been used to study host plant transcriptome dynamics in cyst nematode parasitism. RNA-seq analysis of infected and uninfected *Arabidopsis* roots by *H. schachtii* showed 4206 differentially expressed genes [19]. Transcriptional regulation is not the only gene regulatory mechanism active in host plants during nematode parasitism. As with other cases of plant-pathogen interactions, there is strong posttranscriptional and translational regulatory pressure mediated by microRNAs [20,21]. Moreover, *H. schachtii* affects proteolysis by diminishing the protease activity [22]. This highlights the role of protein stability and turnover in syncytia, as evidenced by transcriptomic data revealing specific and preferential engagement of protein biosynthesis and turnover genes [18,19]. Consequently, specific proteome dynamics are anticipated but have not been verified experimentally on a large scale. A more realistic image of the processes taking place in syncytia would come from proteomic studies. However, due to technical limitations, these studies are still underrepresented among plant nematode-omic projects. Some preliminary results have come from our recent survey on proteins from laser-capture microdissected (LCM) syncytia. Even 50% of proteins preferentially appearing in mature syncytia did not show similar trends at the transcript level [23].

To broaden the proteomic perspective on plant-nematode interactions, this paper analyzes complex proteomic profiles, encompassing 2835 proteins identified in uninfected and infected tomato roots of susceptible and resistant plants carrying the *Hero A* gene, responsible for resistance to *G. rostochiensis*. A comparison of the list of differentially expressed proteins (DEPs) with existing transcriptomes of *G. rostochiensis*-infected tomatoes revealed significant disparities between transcriptomic and proteomic data. Furthermore, putative *Arabidopsis* orthologs of selected DEPs were identified and examined using the *A. thaliana*–*H. schachtii* (beet cyst nematode; BCN) model, which offers biological similarity, extensive host mutant collections, and a variety of bioinformatics and analytical tools for follow-up studies.

## 2. Results

### 2.1. Protein Identification by Mass Spectrometry

**Figure 1:**
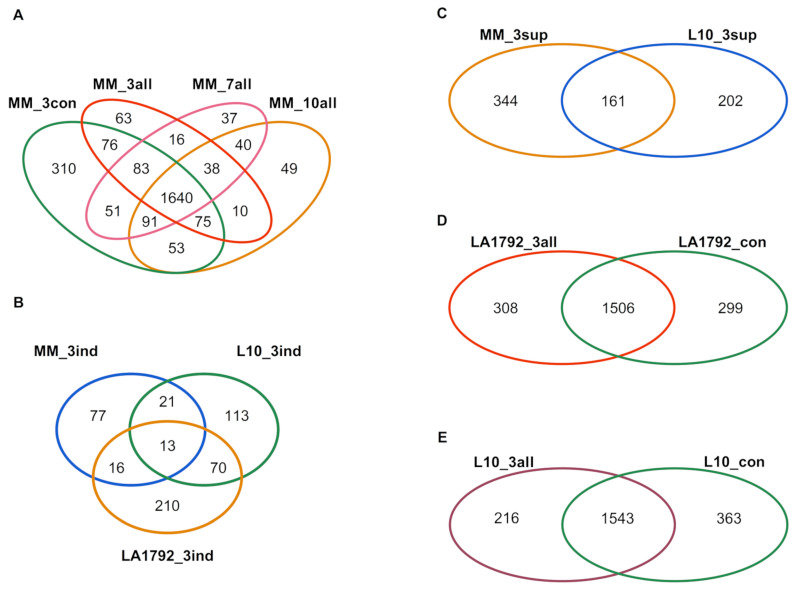
Venn diagrams of proteins identified with high confidence by at least two peptides in roots of resistant and susceptible tomato genotypes after *G. rostochiensis* infection or in uninfected control. (**A**). Uninfected control (MM_3con) and 3, 7, and 10 dpi Moneymaker plants (MM_3all, MM_7all, and MM_10all, respectively). (**B**). MM_3ind, L10_3ind, and LA1792_3ind stand for proteins specifically appearing (induced) in infected tomato roots at 3 dpi from Moneymaker, L10, and LA1792 lines, respectively. (**C**). To visualize proteins suppressed, the uninfected susceptible Moneymaker proteins disappearing after infection (MM_3sup) were compared to a list of suppressed proteins in transgenic line with *Hero A* gene (L10_3sup; the result of comparison visualized on (**E**)). (**D**). Diagram of proteins identified in infected LA1792 roots (LA1792_3all) and uninfected control (LA1792_con). (**E**). Diagram of proteins identified in infected L10 roots (L10_3all) and uninfected control (L10_con).

This study aimed to analyze the proteomes of *G. rostochiensis*-susceptible and -resistant tomato lines in response to nematode infection. We focused on the most developmentally and metabolically dynamic part of the infected root—syncytia, at 3, 7, and 10 dpi of the Moneymaker line and 3 dpi of the LA1792 and L10 lines. These last two lines were analyzed at 3 dpi only due to their resistance to *G. rostochiensis*, which causes a Hypersensitive Response (HR) and syncytium degradation [24]. The nematode larva’s roots invasion, intracellular migration, and syncytium induction and development in the susceptible Moneymaker line were consistent with previous reports [17,25,26]. The mass spectra of root proteomes of infected and control lines were obtained from triplicate root samples. They were matched with the tomato protein sequence database, resulting in 2835 different tomato protein hits, all identified with high confidence by at least two peptides (Supplementary Table S1). The identified proteins varied across various lines, ranging from 1405 in the LA1792 uninfected control to 2162 in the Moneymaker uninfected control. For the samples quality check, we did a PCA analysis which indicated that nematode infection is the most influencing stimulus for proteomic changes followed by genetic variation between LA1792 line and Moneymaker (L10 line is a transgenic Moneymaker and they grouped closely) (Supplementary Figure S1). The distribution of sample replicates was close, confirming their uniformity. Venn analysis of these proteomes revealed interesting qualitative differences (present/absent) between infected and uninfected and resistant and susceptible lines (Figure 1A–E; Supplementary Table S2). For example, 253 tomato proteins appeared explicitly in the infected susceptible cultivar (Moneymaker; 3, 7, and 10 dpi). Among them, 38 proteins were present at all time points, whereas 63, 37, and 49 appeared in a narrow developmental context at 3, 7, and 10 dpi, respectively (Figure 1A). This observation confirms that cyst nematode parasitism is a complex sequence of cellular and molecular processes with limited overlap over time. Interestingly, the proteomic effect of nematode infection on resistant lines L10 and LA1792 was much more complex than in the case of susceptible Moneymaker 3dpi, constituting 216, 308 and 127 newly expressed proteins, respectively (Figure 1A,D,E). We noticed also the strong genetic background dependent specificity among 3 dpi activated proteins (Figure 1B). These numbers, compared with 505, 363 and 299 proteins disappearing upon infection in Moneymaker, L10 and LA1792, respectively, reflect that the negative host plant gene regulation is crucial during nematode larva invasion, migration, syncytium initiation followed by developmental and functional cell reprogramming (Figure 1A,C,D,E).

**Figure 2:**
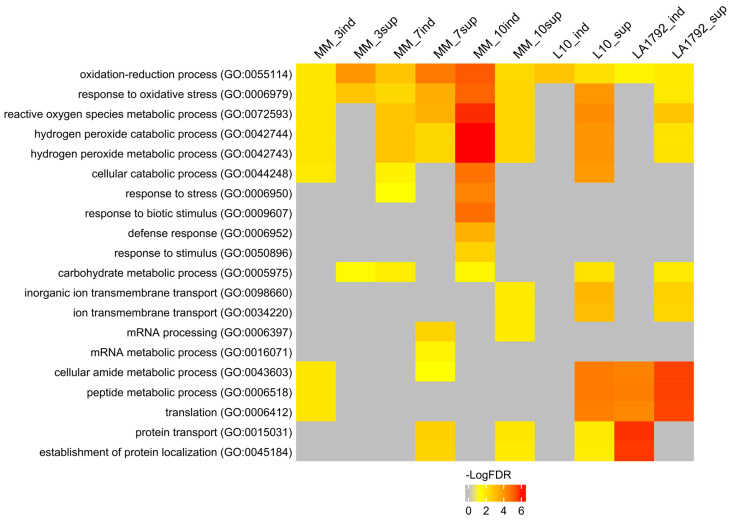
Singular Enrichment Analysis (SEA) of induced and suppressed proteins in nematode-infected tomato roots was performed using the AgriGO toolkit. Significantly enriched biological processes are color-coded according to their false discovery rate (FDR ≤ 0.05). The figure displays 20 selected biological processes. A full list of GO categories describing biological processes, molecular functions, and subcellular localization is provided in Supplementary Table S3, including the number of proteins associated with each GO term and the corresponding FDR value. The protein groups were selected based on Venn analysis (Figure 1A–E). Abbreviations on the figure: MM, L10, LA1792—tomato genotype Moneymaker, Moneymaker with *HeroA* transgene and Aisla Craig *HeroA* introgression line respectively; sup—suppressed proteins, ind—induced proteins (preceding 3, 7 and 10 indicate dpi; L10 and LA1792 are 3dpi only). Number of proteins analysed: MM_3ind—126, MM_3sup—505, MM_7ind—131, MM_7sup—514, MM_10ind—137, MM_10sup—520, L10_ind—216, L10_sup—363, LA1792_ind—308, LA1792_sup—299.

To interpret the presented proteomic dynamics, the gene ontology (GO) of these protein groups was analyzed using the AgriGO toolkit. This toolkit allows for the visualization of Singular Enrichment Analysis (SEA) of specific terms describing protein function, process, and localization (Figure 2; Supplementary Table S3) [27,28]. Ten protein groups were selected based on Venn analysis (Figure 1A–E): induced and suppressed proteins from Moneymaker 3, 7 and 10 dpi (126, 505, 131, 514, 137 and 520 proteins respectively), and induced and suppressed proteins from L10 and LA1792 lines 3 dpi (216, 363, 308 and 299 proteins respectively). Proteins associated with the GO terms ‘oxidation-reduction process’ and ‘response to oxidative stress’ played a leading role in compatible plant-nematode interactions. These proteins were significantly overrepresented in both the induced and suppressed protein groups. Similar but more specific GO categories related to hydrogen peroxide metabolism and catabolism were significantly enriched among induced proteins, with increasing significance at 10 dpi, when the syncytium is already established and functioning. A similar trend was observed for GO categories related to ‘cellular catabolism’, ‘response to stress’, ‘response to biotic stimulus’, ‘carbohydrate metabolism’, and ‘defense response’. Notably, the ‘defense response’ category was not enriched in nematode-resistant tomato lines. Interestingly, among proteins regulated (either suppressed or induced) by nematode infection, the GO categories ‘peptide metabolism’ and ‘protein transport’ were also enriched. This likely reflects the complex interplay between nematode parasitism and programmed cell death, which plays a dual role in both development and defense (Figure 2, Supplementary Table S3).

**Figure 3:**
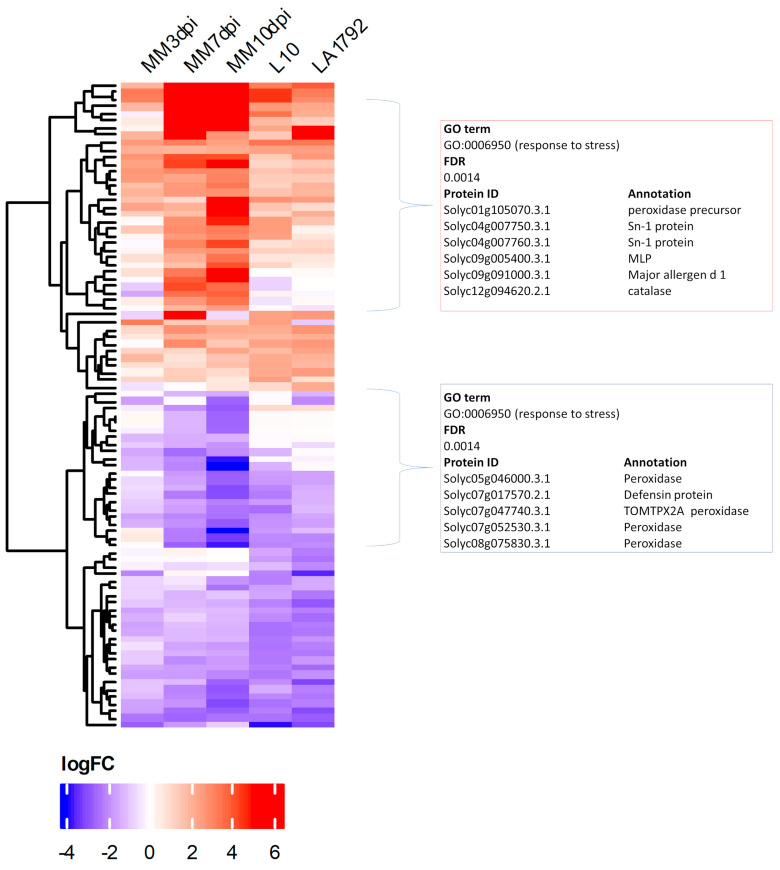
The heatmap of Differentially Expressed Proteins (DEPs) after Label-Free Quantitation presents 90 proteins with fold change log_2_FC ≥ 1 or ≤−1 and FDR ≤ 0.01 from nematode-infected tomato root samples compared to uninfected controls: MM–Moneymaker 3, 7, 10dpi, L10 3dpi and LA1792 3 dpi. The AgriGO analysis resulted in “response to stress” as the most significantly enriched GO term among mostly upregulated and one mostly downregulated proteins. Fold change values, genome indices and AgriGO results are presented in Supplementary Table S4 and Supplementary Figure S3.

Presented above qualitative differences (protein detected/undetected) between the proteomes of infected and uninfected root samples represent only a portion of regulatory dynamics that result in successful syncytium initiation, development and functioning on susceptible plant roots or syncytium initiation and degradation in the case of *Hero A* gene action. Therefore we supplemented our analysis with a quantitative approach—Label-Free Quantitation (LFQ) [29]. Statistical analysis revealed a total of 90 Differentially Expressed Proteins (DEPs) filtered at FDR ≤ 0.01 (volcano plots are in Supplementary Figure S2), including both upregulated (log_2_FC ≥ 1.0) and downregulated (log_2_FC ≤ −1.0) proteins, in the following five pairwise comparisons: infected susceptible Moneymaker 3, 7, and 10 dpi samples vs. uninfected control and analogous infected/uninfected comparisons of resistant L10 and LA1792 lines at 3 dpi (Figure 3 and Figure S3). There are slightly more proteins downregulated in Moneymaker and L10, whereas LA1792 shows the reverse trend, with more proteins upregulated. Similarly, as in the case of qualitative proteome differences, the DEPs were analyzed with the AgriGO toolkit for the enrichment of specific GO terms, resulting in the most significant enrichment of the “response to stress” category among coexpressed genes in both upregulated and downregulated groups (Figure 3). Interestingly, there are more similarities between 3 dpi proteomic dynamics of susceptible and resistant lines than Moneymaker 3 dpi compared to 7 and 10 dpi, indicating that syncytium development and functioning is more complex in terms of gene regulation than activation of hypersensitive response.

### 2.2. Study of the Tomato Orthologs in Arabidopsis

Based on the presented analyses, four proteins potentially essential for host response to nematode infection have been selected for further studies: Apyrase 2 (APY2; Solyc12g098540), Nitrate Reductase 2 (NIA2; Solyc11g013810), gamma-aminobutyric acid transaminase (GABA-T; Solyc12g006470), and AAA-ATPase 1 (AATP1; Solyc05g007470). APY2 protein level increases substantially in Moneymaker at 7 and 10 dpi, while at 3 dpi it is expressed at a low level regardless of genotype and resistance. NIA2 is strongly upregulated in both resistant lines and at 10 dpi in Moneymaker but showed only a slight increase in compatible interaction at 3 and 7 dpi. GABA-T protein is strongly upregulated in all infected lines at all data points. In contrast, AATP1 is strongly upregulated in resistant lines at 3 dpi, stable in Moneymaker at 3 dpi, and slightly upregulated at later stages (Supplementary Figure S3).

Other researchers have experimentally confirmed the selected enzymes’ significant role in plant responses to pathogens. The Apyrases are linked to controlling H_2_O_2_ levels in plants, which is crucial in defense against fungal pathogen attacks [30]. Nitrate reductase 2 is involved in producing nitric oxide, a molecule that plays a significant role in plant immunity [31]. The GABA-T superfamily is involved in the synthesis and metabolism of GABA in plants in response to biotic and abiotic stress [32]. Finally, AAA-ATPase1 is essential in SA-mediated defense responses against blast fungus *M. oryzae* [33].

One of the most valuable experiments to document a given gene role in plant-nematode interaction would use its mutant or overexpressor; however, preparing such lines, especially of non-model species, usually exceeds the timeframe of most projects. Since the desired outcome of many plant-pest interaction studies is the identification of general resistance/susceptibility mechanisms not limited to one plant species, we decided to select putative orthologs of our candidate genes in the *Arabidopsis* genome and work on their mutants. Additionally, *Arabidopsis* is a host for *Heterodera schachtii*, close relative of *G. rostochiensis*. These two host and nematode species are frequently used to confirm solanaceous plant *Globodera* interactions.

**Figure 4:**
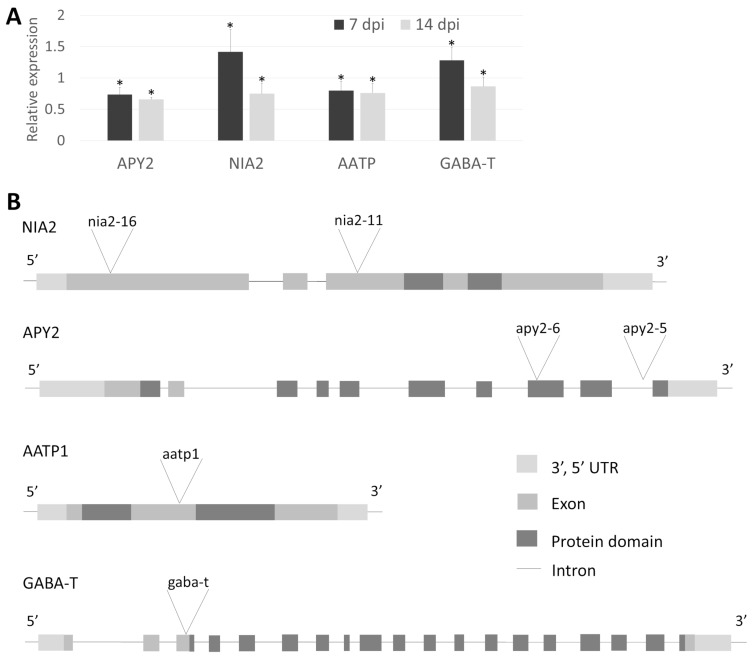
(**A**). Relative expression levels of four *Arabidopsis* orthologue genes at two time points (7 and 14 dpi) after *H. schachtii* infection as quantified by RT-qPCR analysis in Col-0. Each data point represents the average ± SE from three independent biological replicates obtained from pooled samples from three plants. All bars represent statistically significant results, with *p* < 0.05 (*). (**B**). T-DNA insertions localization in *Arabidopsis* genes homologous to tomato candidates selected based on proteomic study. *NIA2* and *APY2* are represented by two independent mutants per gene called *nia2-11*, *nia2-16*, *apy2-5* and *apy2-6*.

The similarity of presumptive *A. thaliana* orthologs ranged from 75% (identical residues) for NIA2 and 74% for GABA-T to 58% for AATP1 and 55% for APY2 (Table 1). All four *Arabidopsis* genes have a similar structure to their tomato counterparts and contain similar functional domains. NIA2 has two oxidoreductase domains (OxR-FAD and OxR-NAD), APY2 has a GDA1_CD39_NTPase domain, AATP1 has AAA_N_dom and AAA+_ATPase domains, and GABA-T has a large, Aminotrans_3 domain. The transcript levels of four selected orthologs were analyzed in Col-0 infected with *H. schachtii* at two time points: 7 and 14 dpi. At 7 dpi, NIA2 and GABA-T transcripts were upregulated, whereas by 14 dpi, all four transcripts were downregulated (Figure 4A). Although the magnitude of these changes was relatively small compared to the original tomato proteome dynamics, they were statistically significant. The observed expression trends also differed between species. However, a comparison of tomato candidates and *Arabidopsis* putative ortholog gene expression profiles in published transcriptomic data (ePlant; http://bar.utoronto.ca) revealed substantial similarity between the two species (Supplementary Figure S4).

**Figure 5:**
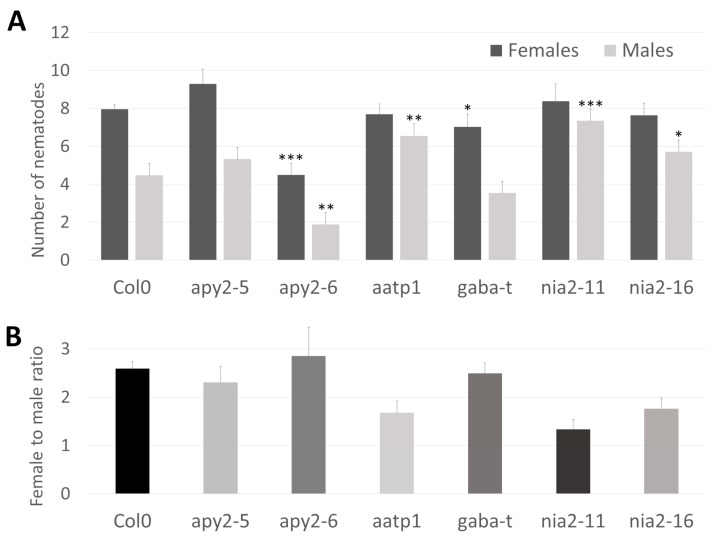
Nematode infection tests. (**A**). Number of male and female nematodes detected on host *Arabidopsis* plants. The lines *apy2-5*, *apy2-6*, *nia2-11* and *nia2-16* represent different insertions within the *APY2* and *NIA2* genes, respectively. (**B**). Female to male ratio for studied mutants. Significance values of the Wilcoxon test were: * *p* ≤ 0.05, ** *p* ≤ 0.005 and *** *p* ≤ 0.0005.

To directly demonstrate the role of the chosen genes in the interaction with parasitic nematodes, we conducted infection assays with corresponding mutants and wild-type plants. Two T-DNA insertion alleles were selected for both *NIA2* (*nia2-11*, *nia2-16*) and *APY2* (*apy2-5*, *apy2-6*). Additionally, single mutants were chosen for *AATP1* (*aatp1*) and *GABA-T* (*gaba-t*) (Figure 4B). Genotyping of *Arabidopsis* mutants confirmed the insertion sites of each allele. *NIA2* mutants (*nia2-16* and *nia2-11*) contain insertions within exons—one near the 5′ end and the other in the middle of the coding sequence. *apy2-6* carries an insertion within the functional domain, while *apy2-5* has an intronic insertion, both located closer to the 3′ end. *aatp1* harbors an insertion between functional domains in the middle of the coding sequence. The *gaba-t* mutant has an insertion within the coding sequence near the 5′ end before the functional domain begins (Figure 4B). The *apy2-6* mutant exhibited the lowest number of both female and male nematodes per plant, highlighting the substantial role of APY2 in nematode parasitism (Figure 5A). Most other mutants showed significant changes in nematode parasitism affecting only one sex: a reduction in females for *gaba-t* and an increase in males for *aatp1*, *nia2-16*, and *nia2-11*. Interestingly, although changes in the female-to-male ratio were observable for *aatp1*, *nia2-16*, and *nia2-11*, they remained statistically insignificant (Figure 5B).

## 3. Discussion

### 3.1. Tomato Proteome Analysis

Transcriptomic profiling has become a routine approach for characterizing biological processes and discovering genes in humans, animals, and plants. Although it reflects only the initial step of gene activation, advancements in methodology (such as RNA-seq and other techniques), the ability to work with small sample sizes (including single-cell cDNA amplification), and the availability of external services and analytical tools at affordable prices have made transcript-level assessment a fundamental—and often the sole—measure of gene function. Meanwhile, several studies indicate that the correlation between transcript and protein quantities is often unclear [23,34,35,36]. This is also the case in most plant-nematode interaction studies. So far, all studies of host plant and *G. rostochiensis* interactions have focused on transcriptome dynamics, and to the best of the authors’ knowledge, the proteome perspective of plant response to this pest was addressed only in their previous report [23].

In this study, nematode-infected and uninfected proteomes of three tomato lines were examined: PCN susceptible Moneymaker, L10, transgenic tomato line containing *Hero A* gene in the Moneymaker genetic background, and LA1792, possessing the complete cluster of *Hero* genes introgressed to a PCN-susceptible Ailsa Craig background. *Hero A* is a tomato gene conferring high resistance to all *G. rostochiensis* pathotypes and partial resistance to *G. pallida* [24]. It represents NBS-LRR resistance genes against plant cyst nematodes [37]. Because of the strong HR of the lines containing *Hero A*, their proteome was surveyed at 3 dpi only, and samples of 7 and 10 dpi were collected from Moneymaker plants only.

At the first step of our analyses, all high-confidence proteins were surveyed in tomato cultivars with diverse responses to pathogens. In comparing qualitative differences (present/absent), we observed an intriguing switch from predominant protein downregulation in compatible interaction to an almost balanced regulatory strategy in the case of resistance gene presence in the same genetic background and inverted proportion in another background (Ailsa Craig; Figure 1A,D,E). This observation may call into question the conclusions of many studies, highlighting the need for caution before generalizing intriguing findings.

A careful analysis of our results confirms the active suppression of plant defense by nematode larvae in compatible interactions. For example, among the 310 proteins uniquely present in the uninfected, susceptible Moneymaker cultivar, numerous defense-related proteins were identified, including four distinct peroxidases and glutathione peroxidase. Peroxidases generate reactive oxygen species, which play multiple roles in biotic stress responses, such as triggering the biosynthesis of defense proteins and inducing stomatal closure. Additionally, TMT1, an S-adenosyl-L-methionine-dependent methyltransferase, likely contributes to glucosinolate metabolism and defense against phytopathogens [38]. In turn, the ARP protein complex known for its role in penetration resistance against fungal invasion in *Arabidopsis*, may have a similar function in resisting nematode invasion in host plants [39]. Meanwhile, the GO analysis of quantitative differences revealed a corresponding reduction in several defense response proteins in susceptible cultivars (Figure 3).

Some of the detected proteins have already been studied in the context of plant-nematode interactions. In barley infected by *Heterodera filipjevi*, a nematode closely related to those in our study, catalase activity was shown to increase [40]. In our proteomic analysis, catalase was upregulated in *G. rostochiensis*-infected tomato lines, both susceptible and resistant (Supplementary Table S4). Other proteins identified in our results have also been validated through enzyme activity studies. Catalases and ascorbate peroxidases are well-known for their role in reactive oxygen species metabolism and plant pathogen responses [40]. Additionally, nitrate metabolism and the role of NIA2 protein have been investigated in the context of host responses to nematode infection. It has been postulated that reactive nitrogen species play a crucial role in plant defense against beet cyst nematodes, helping fine-tune the infected plant’s response to stress induced by these phytoparasitic nematodes [41].

In addition to proteins with a well-established role in defense responses, we focused also on other intriguing protein groups related to carbohydrate metabolism, transport, signaling, and amino acid metabolism. These proteins exhibited different behaviors in susceptible and resistant lines upon infection. Within the carbohydrate metabolism group, beyond a clear complexity shift in the resistant line at 3 dpi, six proteins showed particularly notable changes in regulation: they were induced in the resistant line but suppressed in the susceptible line. Among them were the aldolase-type TIM barrel family protein (Solyc06g081980.1) and β-glucosidase 17 (Solyc08g044510.3). Aldolases are enzymes involved in sugar metabolism, specifically in the breakdown of glucose and fructose during glycolysis and fructolysis. The aldolase-type TIM barrel protein plays a role in carbohydrate metabolism and the pentose phosphate pathway, providing energy for defense responses. Vanholme et al. [42] also demonstrated that aldolase is involved in lignification and phenolic metabolism during stress. Pathogen-induced lignification is a well-documented plant defense strategy [43], and recent studies have shown that Casparian strip lignification plays a role in plant responses to nematode parasitism [44]. Furthermore, lignification during syncytium development is expected to be both spatially and temporally diversified, as anatomical studies have documented lignified secondary xylem formation around syncytia [45]. β-glucosidase 17 belongs to a large family of β-glucosidases, which function in the final phase of cellulose degradation by hydrolyzing cellobiose residues, thereby modifying cell walls in response to pathogen attacks. This process is widely regulated in nematode-infected plant roots [17,46,47]. Additionally, many plants employ direct chemical defenses against herbivore attacks. For instance, low-molecular-weight compounds are stored as biologically inactive pro-toxins in intact tissue and are enzymatically converted into bioactive toxic compounds upon tissue damage by an herbivore. This two-component system prevents auto-toxicity, allowing plants to store defensive molecules without harmful effects. A common activation strategy is the enzymatic removal of a protective glucose group by a β-glucosidase, which may be another mechanism reflected in our proteomic analyses [48].

### 3.2. Selected Mutant Genes Analysis

This study selected and characterized four candidate proteins based on proteomic analyses. The selection criteria were as follows: 1. Presence among proteins with significant quantitative differences to avoid false positives, 2. preference for upregulation upon nematode infection, as loss of function mutants for these proteins are more accessible for testing, 3. existing literature suggesting their involvement in biotic stress responses, and 4. representation of new molecular functions in the context of previous reports on plant-nematode interactions. Due to the lack of easily accessible tomato mutants, the corresponding putative orthologs were selected and analyzed in *A. thaliana/H. schachtii* model system. To which extent the selected *A. thaliana* genes are true orthologues and whether they reflect the same function as in tomato remains an open question.

Apyrase2, upregulated at 7 and 10 dpi in Moneymaker, catalyzes the hydrolysis of phosphoanhydride bonds of nucleoside tri- and di-phosphates [49]. Apyrases were shown to play essential roles in the signaling steps in plant defense responses [50]. Apyrase suppression significantly altered the expression of genes involved in biotic stress responses, leading to increased extracellular ATP and cell wall lignification [50]. As mentioned above, discussing the possible role of host Aldolase in nematode parasitism, the connection of Apyrase2 to the lignification process is symptomatic. Approximately 50% nematode susceptibility reduction in *apy2-6* was the highest among tested *A. thaliana* orthologue mutants (Figure 5A). Its T-DNA insertion eliminates quite a substantial C-terminal portion of the protein (as compared to another tested *apy2-5* mutant, Figure 4B), potentially rendering it nonfunctional or unstable, opening an exciting avenue for further research. Apyrase2 seems essential for later stages of syncytium development and functioning in tomato and *Arabidopsis*, and its disruption seems to efficiently impair cyst nematode parasitism.

In our studies, Nitrate reductase 2 (NIA2) was upregulated, particularly in the resistant lines and at later stages of compatible interaction. Such upregulation pattern may reflect its specific role in cyst nematode parasitism. NIA2 is a key enzyme involved in the first step of nitrate assimilation in plants, fungi, and bacteria [51]. It catalyzes the reduction of nitrate (NO_3_^−^) to nitrite (NO_2_^−^), which is itself reduced to ammonia (NH_4_^+^) by nitrite reductase (NiR) before being assimilated into the amino acids and the nitrogen compounds of the cell [52]. Nitrogen metabolism in plants interacting with nematodes is intriguing [53]. The enchancement of amino acid biosynthesis, may enrich the nematode diet while keeping turgor pressure in syncytia high [54]. What is interesting is that *NIA1* and *NIA2* mRNAs are cleaved by DICER-LIKE 2 (DCL2) RNAse to 22-nucleotide small interfering RNAs (siRNAs), which repress the expression of cognate genes translationally and create a sophisticated regulatory loop [55]. It was suggested that the production of 22-nt siRNAs, particularly from *NIA1/2*, might be essential for plants to adapt to salinity stress and, in the broader view, to other stresses. The efficient repression of *NIA1*/*NIA2* genes could be a common regulatory mechanism when plants encounter environmental stresses that allow them to deliberately switch from growth to defense [55]. The important role of such small regulatory RNAs in plant-nematode interaction was postulated and analyzed [21]. In light of the presented results, the expected reduction of *NIA2* transcript in *Arabidopsis* mutant increased susceptibility slightly but only when male nematodes were counted. Such a result is interesting, but to interpret it, more research has to be done answering questions about the possible compensation of mutation by NIA1, the opposite susceptibility trend when female to male ratio is counted, dependency of mRNA, siRNA, and protein level, and resulting enzyme substrate/product levels.

In the presented results, the GABA-T level was upregulated in all lines and time points upon nematode infection. GABA-T is a transaminase that degrades gamma-aminobutyric acid [GABA] and uses pyruvate or glyoxylate as an amino-group acceptor [56]. GABA is an ubiquitous four-carbon, non-protein amino acid. It has been widely studied in animal central nervous systems, where it acts as an inhibitory neurotransmitter [32]. In plants, it influences many metabolic pathways involved in physiological processes, including biotic stress. GABA production is highly induced in stems of *Jatropha curcas* infected with the Jatropha Mosaic Virus (JMV) and in tomato leaves infected by *Botrytis cinerea* [57]. Proteomic analysis showed significant downregulation of GABA biosynthesis in tomato stems inoculated with highly and mildly aggressive *Ralstonia solanacearum* isolates [58]. GABA activates antioxidant enzymes (peroxidase, superoxide dismutase, and catalase) and limits cell death, which excessive ROS can cause. GABA inevitably accumulates in the host plant in response to bacterial, fungal infection and infestation by invertebrate pests; however, the mechanism of action for GABA appears to differ. For example, higher GABA levels delay the development of insect and root-knot nematode larvae, presumably by disrupting the function of neuromuscular junctions [59,60]. Accordingly, our tests on *A. thaliana* mutants with substantial *GABA-T* transcript reduction showed a slight reduction in susceptibility, suggesting an increase in GABA. Thus, high GABA levels indicate plant resistance to pests and pathogens, whereas GABA-T upregulation in nematode-infected plants potentially reduces GABA concentration, suggesting effective suppression of plant defense. However, slightly weaker upregulation of GABA-T in resistant lines at 3 dpi suggests attenuation of defense suppression evoked by cyst nematodes or that *Hero A* nematode resistance does not involve GABA.

AAA-ATPase1, upregulated in infected roots but more clearly in resistant lines, belongs to the large AAA+ superfamily of ATPases characterized by a conserved catalytic AAA+ module. It occurs in all life forms, including eukaryotes, prokaryotes, and archaebacteria. It is implicated in various cellular activities, including proteolysis, protein folding, membrane trafficking, cytoskeletal regulation, organelle biogenesis, DNA replication, and immune responses [61]. It was shown that multivesicular bodies (MVBs)-localized AAA ATPase LRD6-6 in rice inhibits plant immunity and cell death, most likely through modulating MVBs-mediated vesicular trafficking in rice [62]. In *Arabidopsis*, another AAA+ ATPase domain-containing protein, mitochondrial AAA ATPase (AtOM66), appears to be part of the biotrophic/SA signaling network, and its overexpression stimulates the PCD markers and leaves the plant more tolerant to infection by biotrophic pathogens [63]. Thus MVBs/endosomes-mediated vesicular trafficking may play critical roles in plant immunity and cell death. The strong evidence for such process engagement during nematode feeding structure development on *Arabidopsis* roots with enhanced PCD we presented previously [19]. Unfortunately, the mutant tested showed only a slight effect on *A. thaliana* susceptibility, which is difficult to interpret without further research, including the AtOM66 overexpressor (and other AAA-ATPase family members), the MVBs formation, and PCD. Another intriguing aspect of AAA+ proteins is that they are considered a novel type of molecular chaperone [64]. The presence of the AAA-ATPase domain in Hero A gives an intriguing dimension for further research on this protein-mediated resistance mechanism.

In conclusion, the presented experiments revealed the tomato root proteome and its dynamics in response to PCN infestation for the first time. This study provided a new perspective on the analysis of nematode parasitism and confirmed that syncytium development and functioning require a sequence of specific molecular functions activated or repressed within relatively narrow intervals. Moreover, nematode defense in resistant lines carrying the *Hero A* gene unexpectedly changes numerous groups of proteins, substantially depending on the genetic background. The identified genes—*Apyrase 2* and *Nitrate Reductase 2*—are good candidates for building durable nematode resistance in crops, or at least warrant further detailed study.

## 4. Materials and Methods

### 4.1. Plant Material

L10 is a transgenic tomato line carrying the *Hero A* gene (Solyc04g008120) in the Moneymaker genetic background. LA1792 is a tomato line Ailsa Craig containing an introgressed *Hero* multigene cluster containing the *Hero A* gene [24,65].

Sterile seeds of tomato (*Solanum lycopersicum* L.) cv Moneymaker, and L10 and LA1792 lines were germinated in vitro on 1.5% (*w*/*v*) B5 medium (Gamborg’s basal salt mixture, 2% (*w*/*v*) sucrose, and 1.5% (*w*/*v*) agar, pH 6.2) and grown in Petri dishes for two weeks in the darkness. The shoots were removed before inoculation.

Seeds of *A. thaliana* L. Heynh. ecotype Columbia (Col0) and mutant lines (all in Col0 background), *apy2* (N662568 and N664280), *nia2* (N666652 and N686876), *aatp1* (N683915) and *gaba-t* (N860071) used in experiments were obtained from Nottingham Arabidopsis Stock Center (NASC, Sutton Bonington, UK). They were surface-sterilized in 0.7% NaClO for 5 min and 70% ethanol (EtOH) for 1 min. In the following step, they were rinsed five times in ddH_2_O. 2 seeds were placed on KNOP medium supplemented with 2% sucrose in a 90 mm diameter Petri dish and grown under a light and temperature regime of 8 h light:16 h dark (SD 8:16) and 22:20 °C. The light intensity was 90 μmol m^−2^ s^−1^ [19].

### 4.2. Tomato Roots Infestation with G. rostochiensis Larvae

Cysts of potato cyst nematode (*G. rostochiensis* Woll.) pathotype Ro1 were surface sterilized in 90% (*v*/*v*) ethanol for 15 s following a 10 min incubation in 1.3% (*w*/*v*) sodium hypochlorite. The cysts were washed 3 times in sterile water and rehydrated in sterile potato root diffusates in the dark at 20 °C for one week. The potato root diffusate was made according to Evans [65]. Surface-sterile J2s were used to infect primary tomato roots directly behind the root tip (100–150 J2s per root). The corresponding root positions were marked for non-inoculated controls in separate plates. The plates were sealed with Parafilm and incubated in the dark at 18 °C. At time points 3, 7, and 14 dpi, tomato root segments from both infected (syncytia detected using a stereomicroscope) and uninfected roots were collected and placed in RNase-free tubes (100 mg of fresh weight per sample) and immediately frozen in liquid N_2_ and stored at −80 °C [17]. One hundred root segments (approx. 50 mg) containing syncytia or root segments alone for control were dissected for each replicate, genotype and time point.

### 4.3. Protein Isolation and Mass Spectrometry

To isolate proteins the dissected tissue fragments were mixed with 100 µL of extraction buffer (100 mM Tris, pH 8.5, 150 mM NaCl, 5 mM EDTA, 10 mM DTT, 0.5% Triton X-100, protease inhibitors cocktail, PhosSTOP phosphatase inhibitors, and 8 M urea). After grinding with a tube-fitted pestle and centrifugation of the solid residues, the proteins were precipitated with methanol–chloroform and submitted to proteome analysis. Samples were resuspended in 100 µL of 100 mM ammonium bicarbonate, vortexed before adding 1ul of 1M DTT, and incubated for 30 min at 50 °C. Then, the samples were cooled before adding 2 µL of 1M iodoacetamide and then incubated in the dark at room temperature for 30 min. Subsequently, the samples were digested with 0.5 µL of trypsin (1 µg/µL) for overnight digest. After digestion, samples were resuspended in 10 µL of 5% formic acid and diluted to 50 µL using Milli-Q water. Three independent biological replicates were used for all proteomic analyses, and the sample reproducibility was visualized by a PCA (Supplementary Figure S1).

Peptide mixtures were analyzed by liquid chromatography coupled to tandem mass spectrometry (LC-MS/MS) using an Ultimate 3000 RSLCnano system (Thermo Fisher Scientific, Waltham, MA, USA), online connected to a LTQ Orbitrap Velos Pro (Thermo Fisher Scientific, Waltham, MA, USA). LC buffers were made up to the following: Buffer A (0.1% formic acid in Milli-Q water (*v*/*v*)) and Buffer B (80% acetonitrile and 0.08% formic acid in Milli-Q water (*v*/*v*)). The peptides were initially trapped on an Acclaim Pepmap100 analytical column (C18, 100 µM × 2 cm) and separated on an Easy-Spray PepMap RSLC C18 column (75 µM × 50 cm; Thermo Electron Corp., San Jose, CA, USA). A program for gradient separation was used (mobile phase A: 0.1% FA in Milli-Q water; mobile phase B: 0.08% FA in 80% acetonitrile, flow rate 0.3 µL/min). The gradient elution started at 2% (0–5 min) of mobile phase B, increased from 5 to 35% (6–130 min), then increased to 98% (130–152 min) of mobile phase B, then returned to 2% (153 min) and remained at this state for the next 20 min.

The peptides were analysed on an LTQ-Orbitrap Velos Pro (Thermo Fisher Scientific) mass spectrometer coupled with a NanoACQUITY Liquid Chromatography system (Waters, Milford, MA, USA). Mass spectrometer equipped with nanoESI ion source was running in data-dependent mode. MS scans were acquired by Orbitrap analyzer set to the resolution of 60,000 and mass range 335–1800 Th. Up to 15 most intense ions from each MS scan were selected for fragmentation. Dynamic exclusion was enabled (exclusion duration 45 s). Collisionally Induced Dissociation (CID) (with multistage activation enabled) and the acquisition of MS/MS spectra were performed in the Ion Trap.

### 4.4. Protein Identification and Quantitation

MS raw files were analyzed by the MaxQuant software (version 2.0.3.0) with default settings, and peak lists were searched against the tomato, *G. rostochiensis* and *G. pallida* FASTA database using the Andromeda search engine [62,66]. The output of MaxQuant, especially Label-Free Quantitation (LFQ) values, were processed by Perseus module. Contaminants, proteins from reversed database, proteins identified only by site and proteins with LFQ available in less than two replicates of any sample were filtered out. LFQ values were log(2) transformed. A fixed modification carbamidomethylation (C) and variable modifications, oxidation (M, P) were used. The false discovery rate was set to 1% for proteins and peptides (minimum length of 7 amino acids) and was determined by searching a reverse database. Enzyme specificity was set as trypsin. A maximum of two missed cleavages were allowed in the database search. For matching between runs, the retention time alignment window was set to 30 min and the match time window was 1 min. Significant results were equal and below FDR = 0.01 (Supplementary Table S1). For further analyses only tomato proteomic data were used (the nematode proteins were filtered out). For LFQ, in MaxQuant, the minimum ratio count was set to two (at least in one pairwise LFQ comparison).

### 4.5. RNA Isolation and RT-qPCR

RNA was isolated from pooled samples using a modified Trizol method (Thermo Fisher Scientific, Waltham, MA, USA) according to Pant et al. [67]. The samples were processed in three biological replicates. Briefly, 100 mg of frozen tissue were ground in liquid N_2_ and incubated in Trizol reagent with 100 μL Plant RNA Isolation Aid (Thermo Fisher Scientific, Vilniaus, Lithuania), 0.5% (*w*/*v*) N-lauryl sarcosine sodium salt, 3 mM β-mercaptoethanol, and 5 mM EDTA. The Ambion Plant RNA Isolation Aid (Life Technologies, Carlsbad, CA, USA) was used to remove polysaccharides during phenol extraction. Next, three phenol/chloroform and two chloroform extractions were performed. RNA was precipitated in the presence of glycogen using 1.25 vol. of ethanol and 0.5 vol. of 0.8 M sodium citrate in a 1.2 M sodium chloride solution. The precipitated RNA was washed with 80% (*v*/*v*) ethanol, air-dried, and dissolved in RNase-free water.

Total RNA (1 μg) was reverse transcribed using random hexamer primers following the manufacturer’s protocol for the QuantiTect Reverse Transcription Kit (Qiagen GmbH; Hilden; Germany).

Real-time PCR cycling conditions were as follows: 5 min denaturation at 95 °C and 40 cycles of amplification (15 s at 95 °C, 30 s at 58 °C, and 30 s at 72 °C). Relative expression levels were calculated using the expression of *actin 2* as an internal reference, according to the 2^−ΔΔ*C*t^ method [68]. Significant differences in expression compared to the control were revealed using the REST tool [69]. Product melting curves were generated following PCR to ensure the purity of the amplification products. A list of all primers used is included in the Supplementary Table S5. The RT-qPCR data were analyzed using ANOVA (*p* < 0.05). The analyses were performed with two reference genes, for *Arabidopsis*, these were the UBQ5 (At3g62250) and UBP22 (At5g10790) genes. In tomato, it was SAND (Solyc03g115810) and RPL8 (Solyc10g006580).

### 4.6. Tomato Orthologues Analysis in Arabidopsis

Genes coding for proteins essential for tomato response to the nematode infection were selected based on their differential expression pattern and their orthologues in *Arabidopsis* discovered using blastn at NCBI server [https://blast.ncbi.nlm.nih.gov] accessed on between 1 and 30 April 2020. Blastn was used as a discontinuous megablast (word size 11; match/mismatch scores 2, −3; gap cost existence: 5, extension: 2; database refseq_rna; organism taxid: 3702). T-DNA insertional mutants were obtained from NASC, propagated in the phytotron, and genotyped to test homozygosity. For genotyping, specific primers were selected using the T-DNA Express: *Arabidopsis* Gene Mapping Tool (http://signal.salk.edu/cgi-bin/tdnaexpress, accessed on 20 May 2021). A list of all primers used is included in the Supplementary Table S5.

### 4.7. Functional Protein Analysis

The GO ontology classification and Singular Enrichment Analysis (SEA) were performed using the AgriGO tool on the web server (http://systemsbiology.cau.edu.cn/agriGOv2/index.php accessed on 23 November 2024) with the following options: organism: *Solanum lycopersicum*, with reference transcript ID ITAG 4.0. GO allowed the classification of infested tomato proteome into different groups.

### 4.8. Nematode Infection Assay

*H. schachtii* Schmidt cysts were harvested from in vitro stock cultures produced aseptically on white mustard (*Sinapis alba* cv. Albatros) roots grown on 0.2 concentrated KNOP medium. The hatching of juveniles was stimulated by incubating the cysts in 3 mM ZnCl_2_ [70]. Second-stage juveniles (J2s) were collected 6–7 days later, sterilized in 0.05% HgCl_2_ for 5 min, and immediately washed five times in distilled H_2_O. Fourteen-day-old *Arabidopsis* plants were inoculated with 80–100 J2s under sterile conditions. Inoculated plates were kept in the dark for 24 h and transferred into a growth chamber under short day (SD) 8:16 photoperiods. The experiments were repeated three times with 10 plants per genotype in one replicate.

The numbers of males and females per plant, the sizes of syncytia, and the associated female nematodes were counted and measured at 14 dpi. For each line, 50 syncytia associated with females were randomly selected and photographed using a Leica M165C stereomicroscope (Leica Microsystems, Wetzlar, Germany) equipped with a Leica DFC 425 digital camera. The syncytia and females were outlined using the Leica Application Suite software (version 3.8). The individual measurements were used to calculate the average size of each syncytium and female. Data were analyzed using a non-parametric Mann—Witney U test with an adjusted *p* ≤ 0.05 for significance.

Root transcriptome level for selected genes in tomato and *Arabidopsis* was confirmed using the ePlant server (http://bar.utoronto.ca/eplant/, accessed on 11 May 2024) [71].

## Figures and Tables

**Table 1:** Orthologs in *Arabidopsis* for selected tomato genes.

Protein Name	Tomato Gene ID	*Arabidopsis* Gene ID
Apyrase2 (Apy2)	Solyc12g098540.2.1	AT5G18280
Nitrate reductase 2 (NIA2)	Solyc11g013810.2.1	AT1G37130
AAA-ATPase 1 (AATP1)	Solyc05g007470.4.1	AT5G40010
Pyridoxal phosphate (PLP)-dependent transferases superfamily protein (POP) (GABA-T)	Solyc12g006470.2.1	AT3G40010

## Data Availability

Data is contained within the article or Supplementary Materials. The original mass spectrometry files and LFQ results file are deposited in Zenodo repository under the following links, respectively: https://zenodo.org/doi/10.5281/zenodo.13319492, published on 14 August 2024 and https://zenodo.org/doi/10.5281/zenodo.13354858, published on 21 August 2024.

## Supplementary Materials

The following supporting information can be downloaded at: https://www.mdpi.com/article/10.3390/ijms26062823/s1.
