# Supplementary material for: Proteomic Dynamics in the Interaction of Susceptible and Resistant Tomato Cultivars and Potato Cyst Nematodes
Source: Int J Mol Sci. 2025 Mar 20;26(6):2823. doi: 10.3390/ijms26062823 (PMC11943225; doi:10.3390/ijms26062823)
Supplement: Supplementary file 1 [file ijms-26-02823-s001.zip › Supplementary Figure S4.pdf]

To confirm expression of selected orthologs in Arabidopsis roots, ePlant server was used

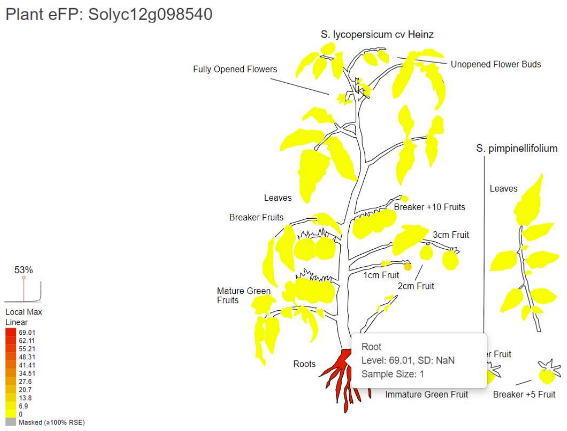

Solyc12g098540 (APY2)

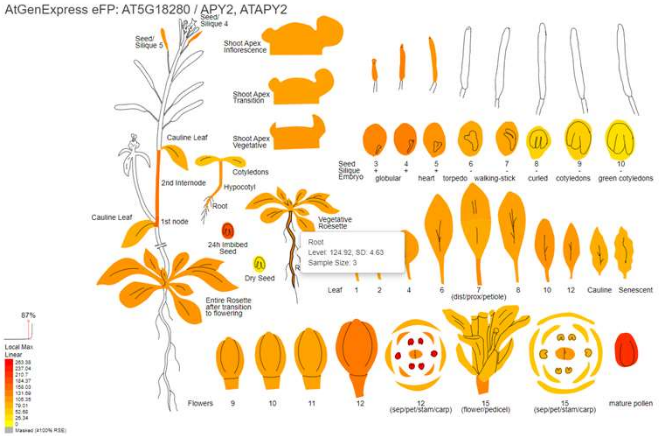

AT5G18280 (APY2)

https://bar.utoronto.ca/eplant/

To confirm expression of selected orthologs in Arabidopsis roots, ePlant server was used

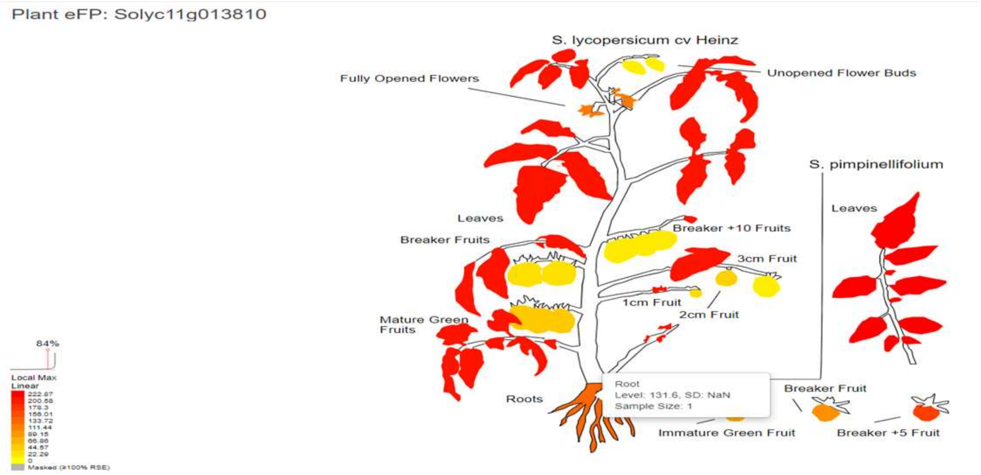

Solyc11g013810 (NIA2)

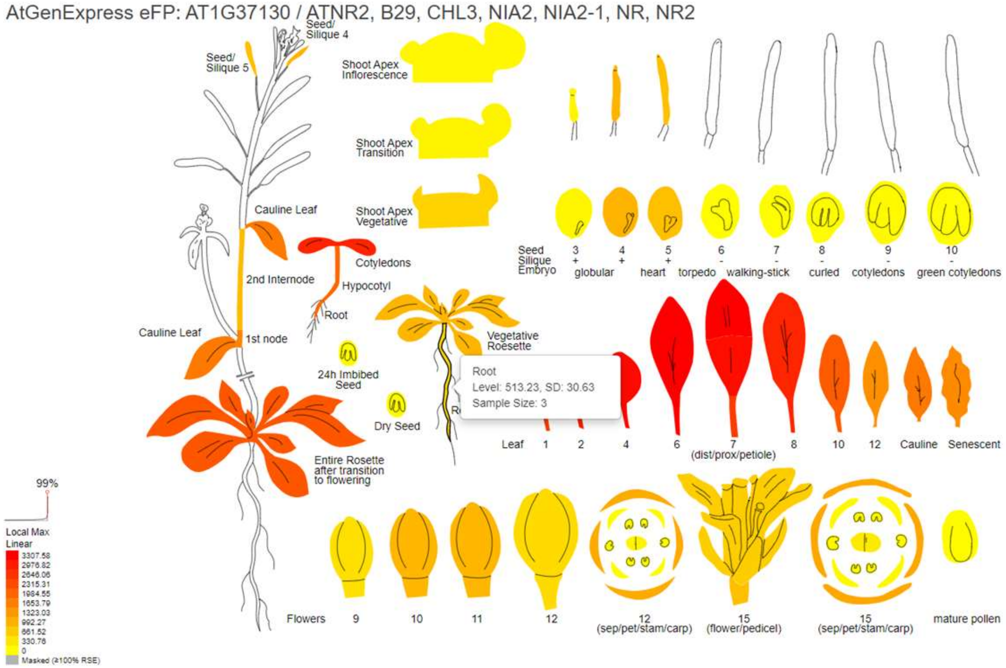

AT1G37130 (NIA2)

https://bar.utoronto.ca/eplant/

To confirm expression of selected orthologs in Arabidopsis roots, ePlant server was used

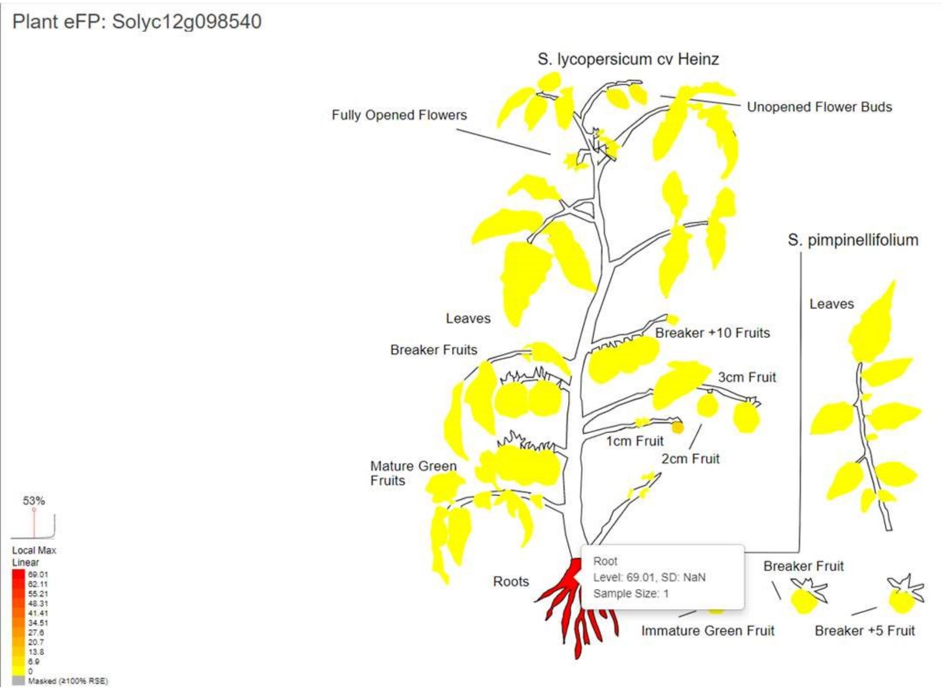

Solyc05g007470 (AATP1)

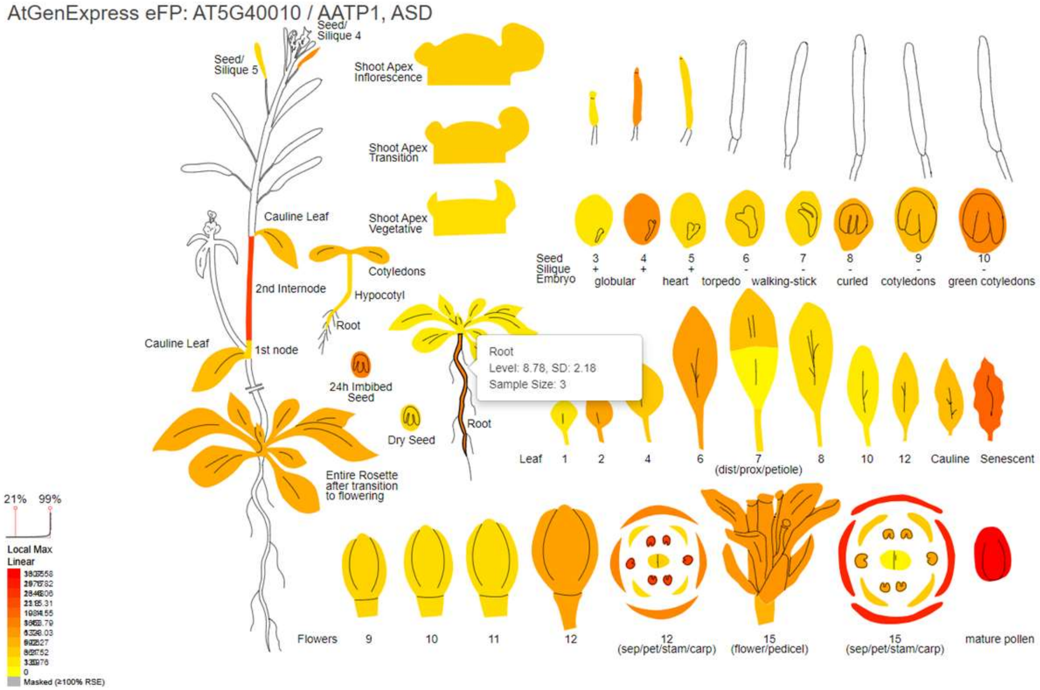

AT5G40010 (AATP1)

https://bar.utoronto.ca/eplant/

To confirm expression of selected orthologs in Arabidopsis roots, ePlant server was used

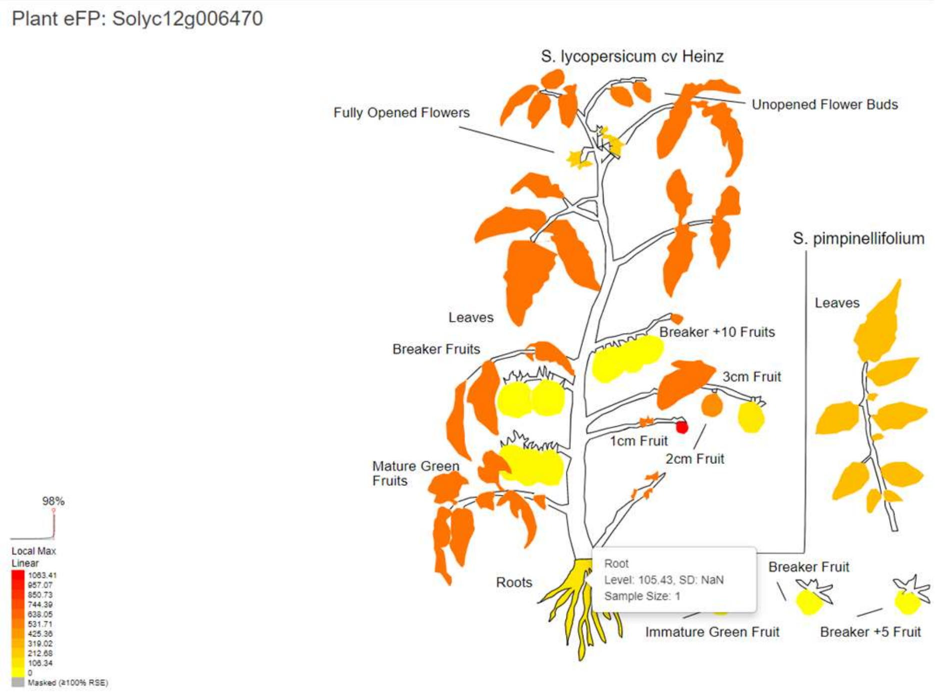

Solyc12g006470 (GABA-T)

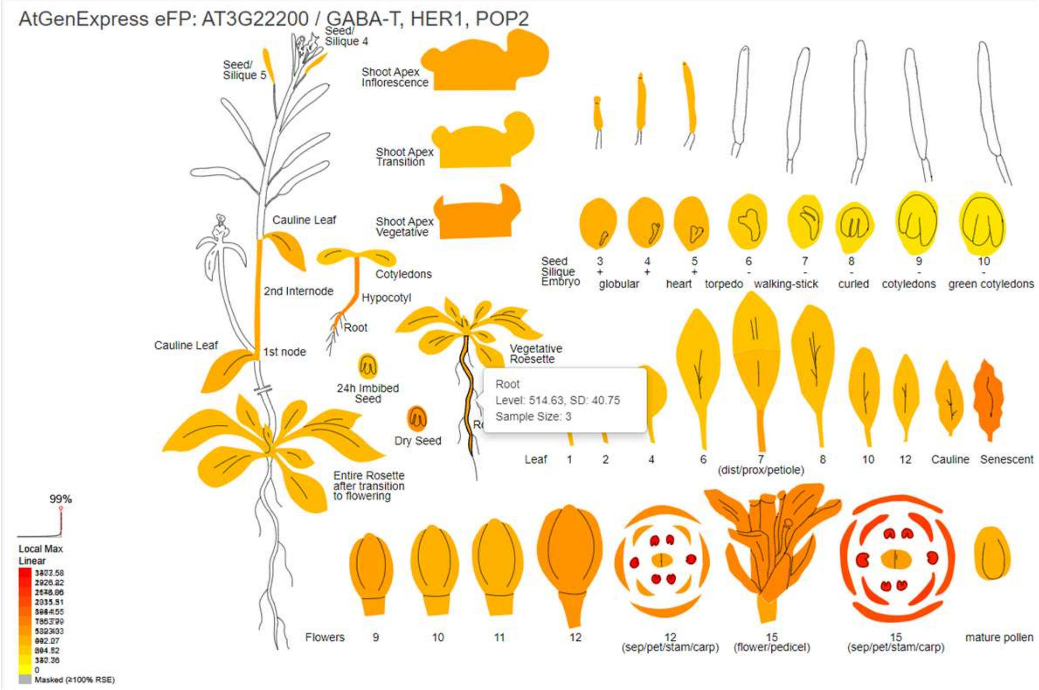

AT3G22200 (GABA-T)

https://bar.utoronto.ca/eplant/
