# Supplementary figures and images for: Proteomic Dynamics in the Interaction of Susceptible and Resistant Tomato Cultivars and Potato Cyst Nematodes
Source: Int J Mol Sci. 2025 Mar 20;26(6):2823. doi: 10.3390/ijms26062823 (PMC11943225; doi:10.3390/ijms26062823)

# Total proteins

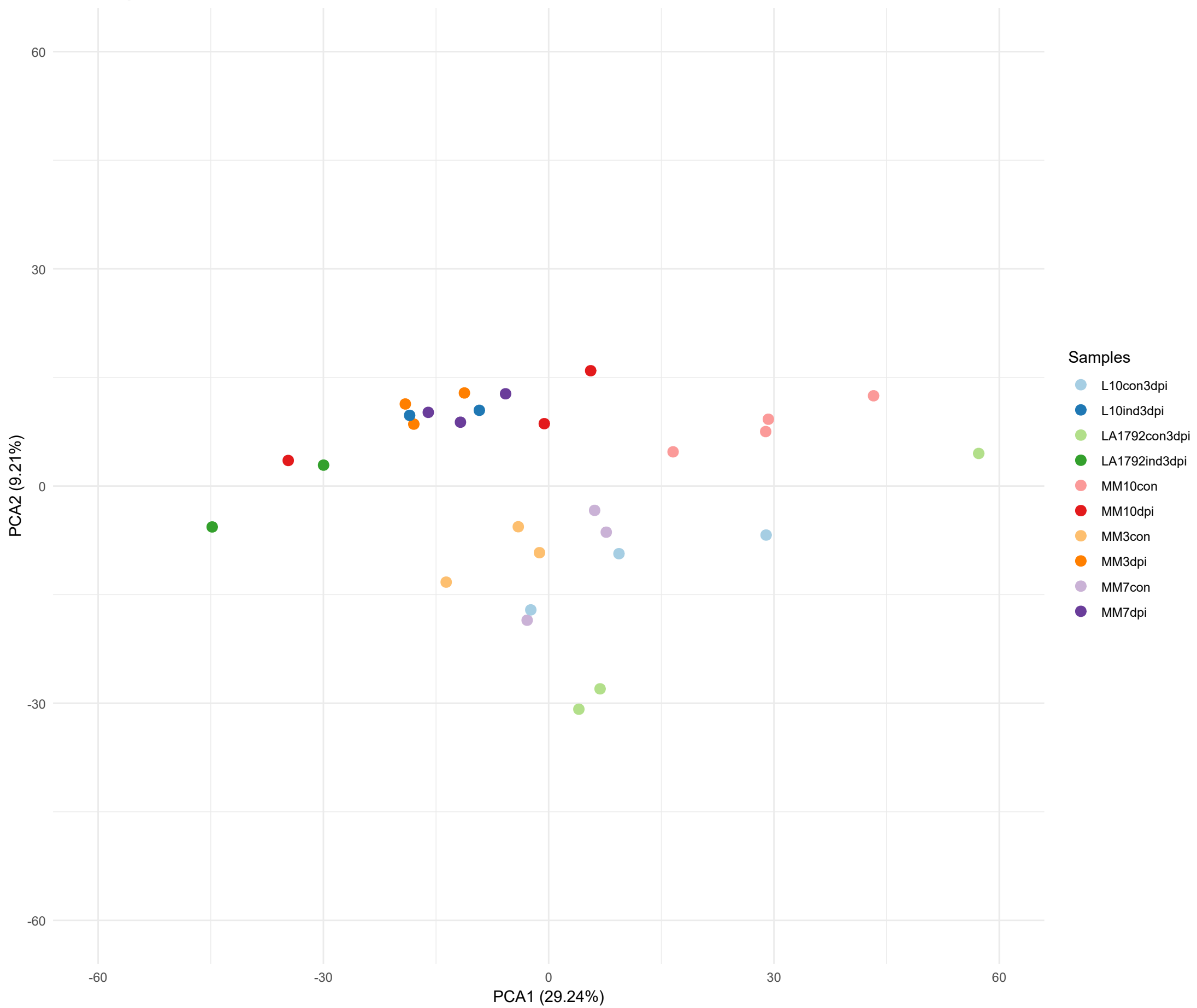

Supplement: Supplementary file 1 [file ijms-26-02823-s001.zip › Supplementary Figure S1.pdf]

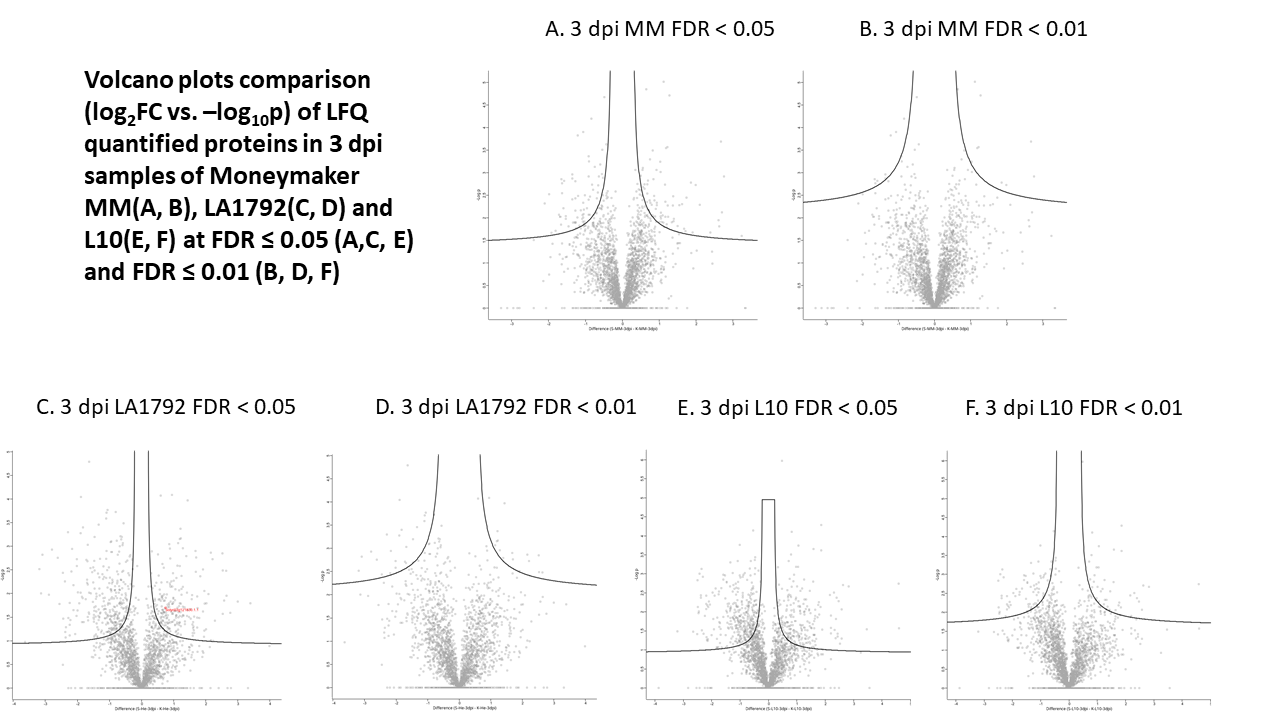

Supplement: Supplementary file 1 [file ijms-26-02823-s001.zip › Supplementary Figure S2.tif]

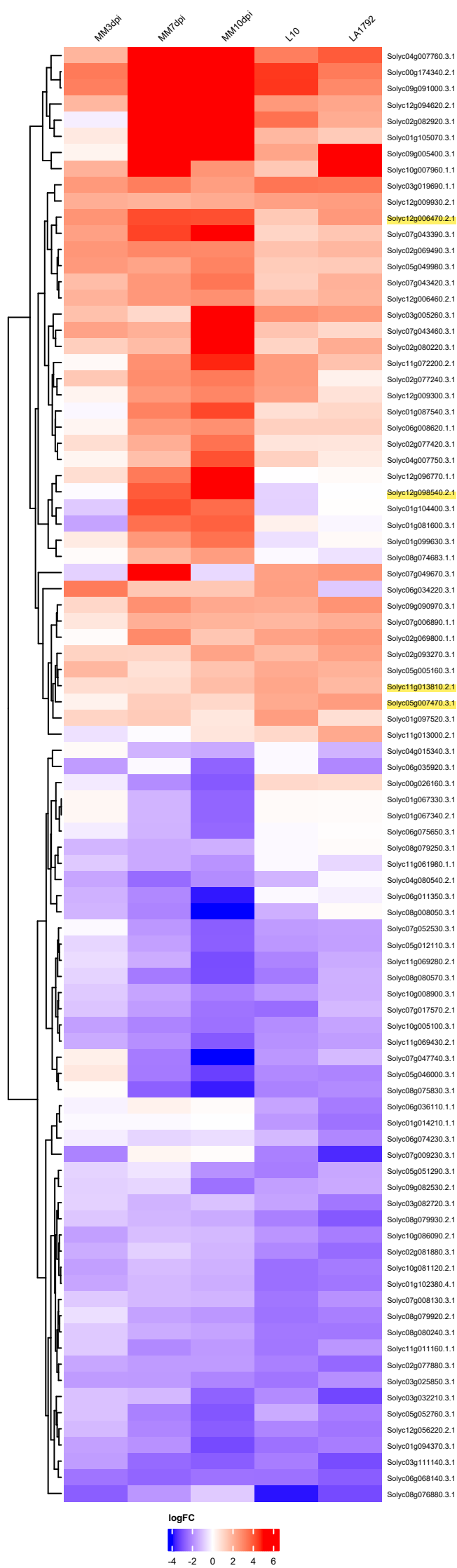

Supplement: Supplementary file 1 [file ijms-26-02823-s001.zip › Supplementary Figure S3.pdf]
